# The importance of modeling epileptic seizure dynamics as spatio-temporal patterns

**DOI:** 10.3389/fphys.2012.00281

**Published:** 2012-07-17

**Authors:** Gerold Baier, Marc Goodfellow, Peter N. Taylor, Yujiang Wang, Daniel J. Garry

**Affiliations:** ^1^DTC Integrative Systems Biology, Manchester Interdisciplinary Biocentre, The University of ManchesterManchester, UK; ^2^Centre for Interdisciplinary Computational and Dynamical Analysis, School of Mathematics, The University of ManchesterManchester, UK

**Keywords:** computational modeling, electroencephalogram (EEG), epilepsy, heterogeneity, spatio-temporal patterns

## Abstract

The occurrence of seizures is the common feature across the spectrum of epileptic disorders. We describe how the use of mechanistic neural population models leads to novel insight into the dynamic mechanisms underlying two important types of epileptic seizures. We specifically stress the need for a spatio-temporal description of the rhythms to deal with the complexity of the pathophenotype. Adapted to functional and structural patient data, the macroscopic models may allow a patient-specific description of seizures and prediction of treatment outcome.

## Epileptic seizure dynamics

Epilepsy is a chronic condition, characterized by acute seizures with unpredictable onsets. The enormous variability of seizures reflects a multitude of genetic and environmental influences (Engel, 2001). Epileptic seizures are defined as “a transient occurrence of signs and/or symptoms due to abnormal excessive or synchronous neuronal activity in the brain” (Fisher et al., 2005). It is generally assumed that clinical symptoms are a consequence of abnormal macroscopic activities depending on their occurrence and locations within the brain. These activities lead to specific features in the electroencephalogram (EEG) which are strongly correlated with clinical symptoms of seizures.

The phrase “excessive or synchronous neuronal activity” in the definition of seizures refers to neuronal activity of large populations of neurons, generally implying that this activity is observable on the macroscopic scale. Epileptic seizures are thereby defined as the result of large-scale neural network activity rather than the activity of individual neurons or small neuronal circuits. The collective nature of the abnormal activity renders it macroscopically observable, e.g., in the EEG. The inclusion of both “excessive and synchronous” activity refers to either strong local potential changes or a high degree of correlation between neighboring potential changes. How these changes correlate with rate and synchrony of neural firing is unclear. For example, parallel measurements of the firing of more than one hundred individual neurons during clinical seizures in humans showed complex changes in neuronal spiking activity including decreased firing rate and decreased synchrony (Truccolo et al., 2011). Seizure discharges are specific macroscopic spatio-temporal activity patterns for which one needs to have a specific (genetic and/or environmental) predisposition. For a deeper understanding of the seizure activity itself, we focus on the specific macroscopic pattern that is the cause of its clinical appearance.

Epileptic seizure dynamics typically takes the form of regular or irregular temporal rhythms in the EEG. A rhythm is a repetitive event that is identified by visual inspection as standing out from normal background activity and being distinct from known physiological EEG rhythms like the occipital alpha rhythms. Epileptic waveforms comprise both linear (sinusoidal) and nonlinear rhythms and the degree of regularity varies strongly between seizure types. As one assumes that these rhythms arise from collective neuronal activity, they are considered to have a strong deterministic (explained) component rather than being solely due to random fluctuations. They should thus be described in terms of nonlinear dynamics, specifically as short realisations of low-dimensional attractors (Lopes da Silva et al., 2003; Lytton, 2008). However, the exact nature of the transient dynamics is unknown at present. A second feature is the spatial distribution of rhythms during the course of a seizure. The study of spatio-temporal patterns is well established in physics (Cross and Hohenberg, 1993) but an application of the concepts to the study of seizure dynamics is not straightforward. Many of the existing models assume spatial homogeneity whereas in epilepsy one almost certainly deals with significant spatial heterogeneity. In particular, for generalized seizures, source localization methods provide evidence for localized onset and differentiated spatial distribution of epileptiform activity (Westmijse et al., 2009; Bai et al., 2010). In focal-onset seizures a localized region is thought to be responsible for the generation of abnormal seizure rhythms (Rosenow and Lüders, 2001). Therefore, both temporal and spatial features are important for a detailed description of clinical seizure rhythms.

## Neural mass modeling

A generic and quantitative description of seizure dynamics in terms of spatio-temporal patterns is provided by neurophysiologically derived neural population models (neural mass models). The mathematical derivation of these models and the explicit equations are beyond the scope of the present perspective but can be found in the literature, see (Deco et al., 2008) for a detailed review. The neural mass models start with a description of the dynamics of neural populations, where the variables are derived as macroscopic representations of ensembles (populations) of similar neurons (Friston, 2008). For the formal description of a single population, either one or two-variable differential equations can be used but more complex equations with more biophysical detail are also available. Based on a number of assumptions, these models then allow the investigation of the interaction between neural populations and explain macroscopic dynamics as observed in, for instance, the EEG. They thus combine a mathematical formalism based on mechanistic neurophysiological knowledge with output that serves to represent clinical observables. Graphical schemes of the involved neural populations and their interactions are given for individual models, e.g., (Wendling et al., 2002; Breakspear et al., 2006; Suffczynski et al., 2006). A body of literature exists on the successful application of these macroscopic models to deal with a variety of phenomena but we focus here on the contribution of neural mass modeling of epileptic seizure dynamics. We describe typical EEG features of two important seizure types and highlight the contributions of neural mass modeling to explain seizure activity as emergent from the interaction of neural populations.

## Generalized seizures

**Figure 1:**
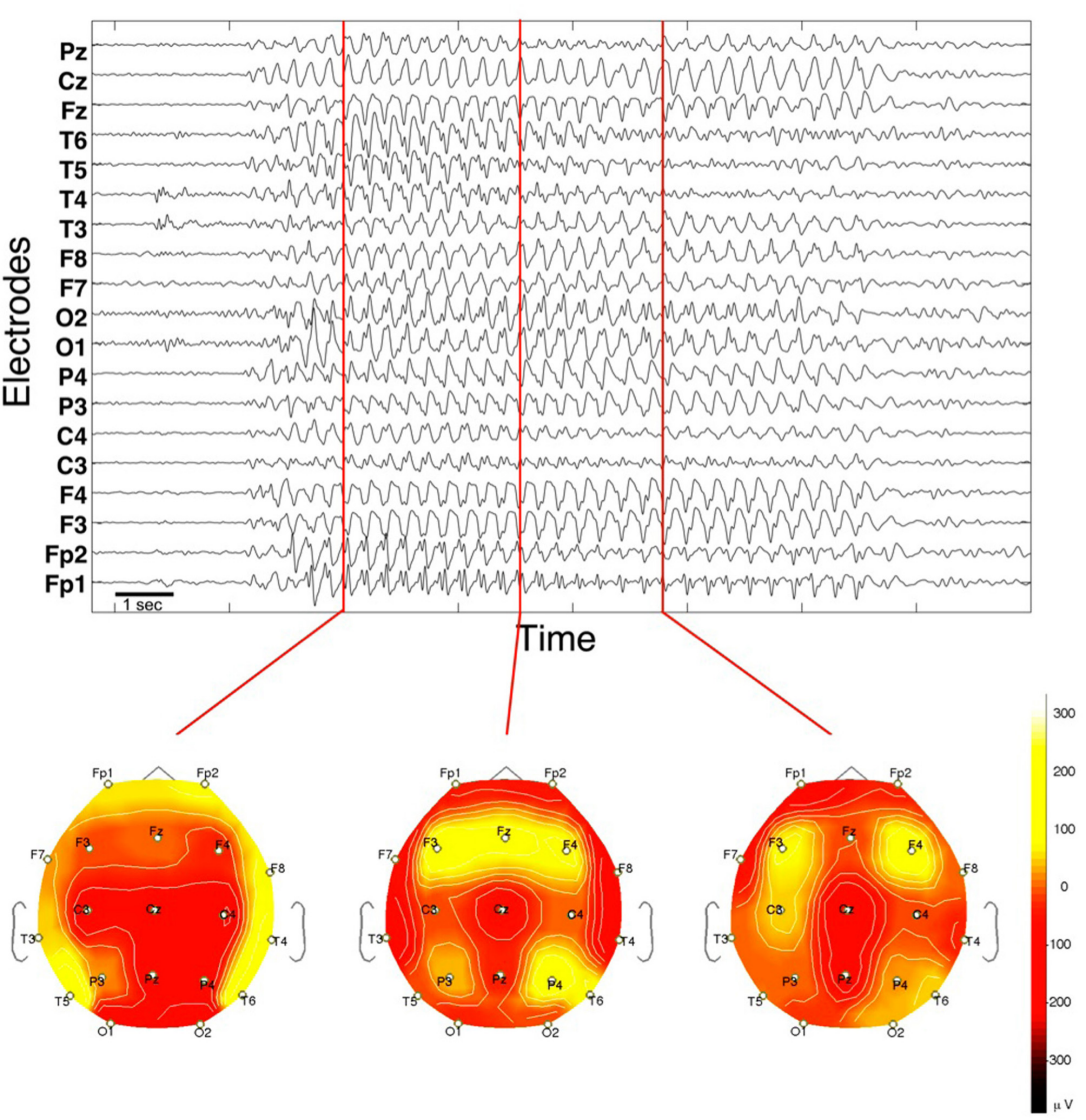
**EEG of a spontaneous absence seizure in a pediatric patient.** Potentials from standard surface electrodes are plotted against time. Horizontal axis spans about 16 s. Below are three topographic potential mappings projected on the scalp (seen from above).

The first type is the generalized absence seizure. The EEG recording of an absence seizure in Figure 1 shows an epileptic rhythm occurring for about 10 s in recordings from surface EEG electrodes. The seizure epoch has a dominant frequency at about 3 Hz and is recognizable by the comparatively large amplitude in all signals. The frequency is consistent across channels, with a trend to decrease toward the end. The most typical feature in absence seizures are the spike-and-wave discharges (SWD). These can best be seen in e.g., prefrontal channels Fp1 and Fp2, while other channels may lack the “spike” component (e.g., channel Cz). Other channels appear to have a more complex waveform (e.g., channel T5 during the first third of the seizure). During the course of the seizure, most channels appear to undergo slow transformations of the waveform. The most obvious spatial characteristic is that the epileptic rhythm is recorded from essentially all locations, and that the degree of correlation (or, more generally, interrelation) is higher during the seizure when compared to pre- and post-seizure periods. Topographic mappings of the spatial voltage distributions are shown for three distinct time points at the bottom of Figure 1.

A number of the dynamical features mentioned have been addressed in neural mass modeling studies. For instance, competing but plausible hypothesis have been advanced for the sudden transition in and out of the seizure state. The first is based on the notion of bifurcation. A bifurcation describes a change in the qualitative behavior of a dynamical system while the setting of the system is varied. In a mathematical model, bifurcations are caused by changes of parameter values. Physiologically this implies some change of e.g., connection strength between or external input to neural populations. Bifurcation theory is then used to characterize and classify these transitions between qualitative states. These states can be identified with, for example, normal and epileptic rhythms (Taylor and Baier, 2011; Wang et al., 2012).

For the explanation of the appearance of SWD in typical absence seizures, a system of interacting thalamic and cortical neural populations was explored (Marten et al., 2009). This study suggested a saddle-node of limit cycle bifurcation. One of the implications of this finding is that a specific change of state is required each time the brain undergoes a transition into the seizure. Alternatively, it was proposed that the dynamical system is in a state of so-called bistability and no parameter change is required for the transition to occur (Lopes da Silva et al., 2003). Physiologically this means that a state of normal brain function and a state of (potentially permanent) epileptic discharges coexist. In a healthy individual these states would be widely separated, however, and the likelihood of seizure would tend to zero. In children with absence epilepsy, the two states would be less separated such that random fluctuations of brain activity in the background state would occasionally lead to a switch to the epileptic state with its characteristic rhythm. A second (equally random) perturbation, or some other mechanism, would then result in the return to the state of normal activity. Both the parameter-dependent and the noise-induced transitions can be explained by a single neural mass model in the vicinity of a saddle-node of limit cycle bifurcation (Marten et al., 2009).

Presently, there are no animal studies that provide direct evidence for either a slow temporal modulation of the state (as implied by a parameter change) or the coexistence of the normal and the epileptic state. However, statistical analysis of long-term recordings has given some support for the latter hypothesis (Suffczynski et al., 2006). In addition, it has been argued that using the temporal variation of the waveform in individual patient recordings, a time-dependent model fit might be used to derive knowledge about an underlying change in physiological conditions (Nevado-Holgado et al., 2012). This could prove a crucial step as patient specific traits have also been observed by other authors (Sadleir et al., 2006; Möller et al., 2008).

A main feature of absence seizures is that they can be picked up from many locations on the scalp and that their electrographic onset is comparatively sudden on most locations of the scalp. The apparent strong correlation between the EEG channels during seizures led modeling researchers to assume that the dynamics is homogeneous in space and that spatial effects can be discarded. However, imaging data (e.g., from combined EEG-fMRI studies) consistently point to a heterogeneous situation across the neocortex and specifically to regions in the brain that can be associated with seizure onset (Möller et al., 2008; Westmijse et al., 2009; Bai et al., 2010). A recent modeling study started to address such features by assuming a small cortical area composed of coupled compartments with heterogeneously distributed parameters across the compartments (Goodfellow et al., 2011). It was found that while the homogeneous model showed a bistability between normal and epileptic state (as proposed previously), the assumption of a heterogeneous medium led to the detection of a new dynamical state. In this new state the dynamics remain in the background activity for most of the time, but autonomously bursts into periods of spike-wave dynamics. Dynamically, this state belongs to the category of spatio-temporal intermittencies and it suggests that seizure dynamics can be spontaneous and self-terminating even in the absence of random fluctuations. Importantly, in contrast to the previous models, the intermittency model also explains the sudden reorganization of correlations between locations: the correlation between sites in the background activity is low due to coupling between sites, while during the seizure state the correlation is high between most but not necessarily all locations. Additionally the model predicts so-called “micro-seizures”, transient spike-wave dynamics in single locations that do not to lead to the collective appearance of seizure dynamics. Micro-seizures have been reported from micro-electrode recordings in humans with partial seizures (Stead et al., 2010) but it is not yet known whether they can occur in human patients with typical absence.

## Focal-onset seizures

**Figure 2:**
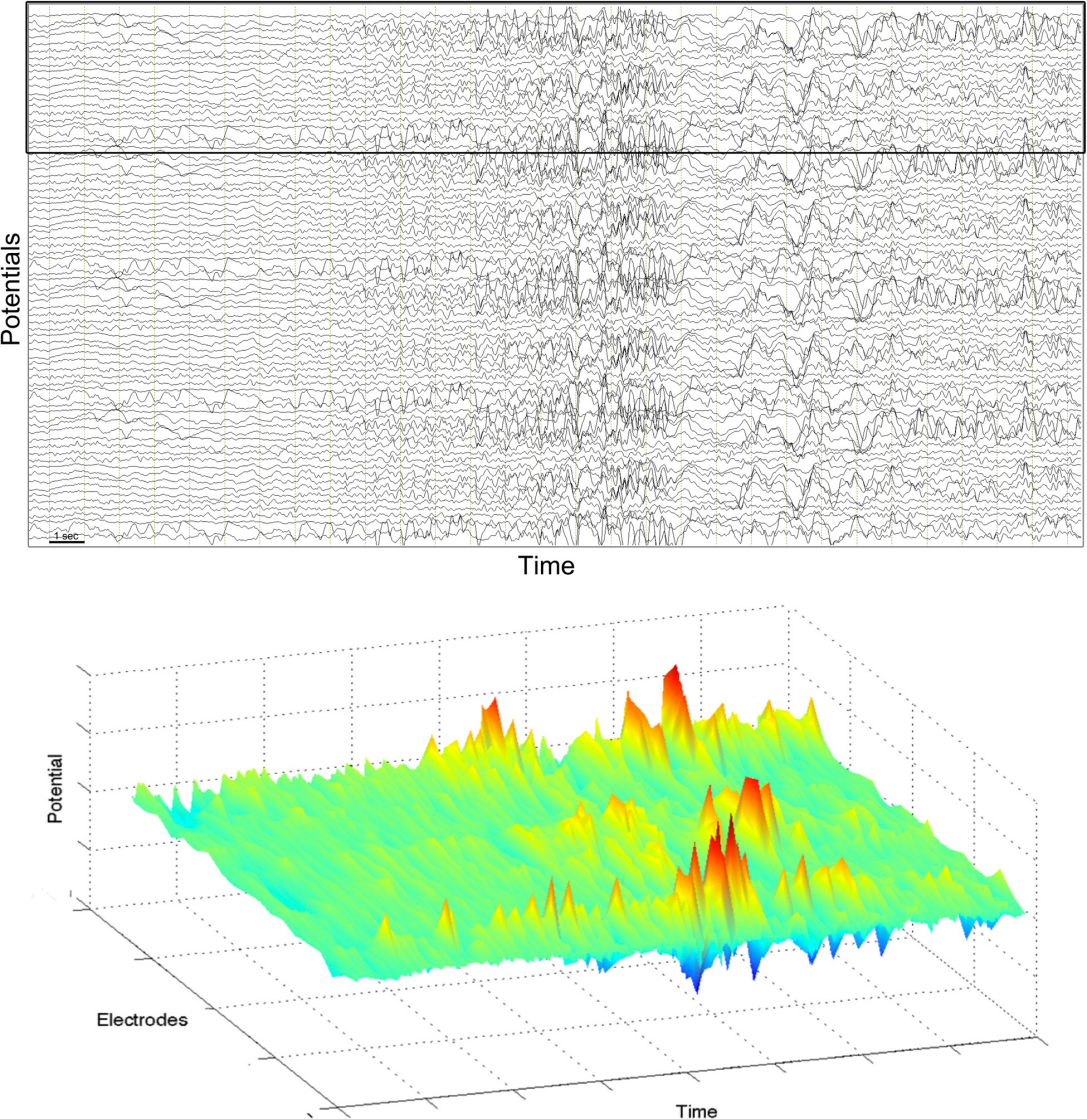
**Electrocorticogram of a partial seizure in an adult patient.** Top: potentials from 78 grid electrodes are plotted against time. Horizontal axis spans about 30 s. Bottom: pseudo-3D plot of 20 electrodes indicated by black frame in the top figure.

The second type of seizure are the focal-onset seizures, where the epileptic activity initiates from a focal region and successively spreads to other regions. Typically, different frequency rhythms are involved in the course of a single seizure (Blume et al., 1984). The frequency can vary continuously or abruptly, and qualitatively different waveforms can often be discerned. Figure 2 shows an example from a focal-onset seizure that originated in the neocortex. This recording is an electrocorticogram, where electrodes are placed on the grey matter of the cortex in a grid covering a few square centimeters. Therefore the spatial resolution in the recording is higher than in the EEG recording in Figure 1. The focal-onset seizure starts with a slow rhythm which is only visible in a few contacts, speeds up (approximately near the middle of the figure), increases in amplitude and starts spreading to neighboring locations. There is a loss of rhythm for about 2 seconds before a new, slow rhythm resumes (last third of the figure). Note that the contacts with a slow rhythm at the end of the recording are not the same as those that showed the initial rhythm. The bottom part of the figure shows a pseudo-3D representation of the corresponding spatio-temporal pattern.

In neural mass modeling the large amplitude rhythms of partial seizures were accounted for by large-amplitude oscillations resulting from a saddle-node on invariant circle bifurcation (Grimbert and Faugeras, 2006). Different frequencies of rhythms and different waveforms can be found within the oscillatory region of the model. To explain the succession of rhythms in a specific type of epilepsy (mesial temporal lobe epilepsy), parameters were fitted to individual segments of the rhythm such that optimal agreement with the data was obtained (Wendling et al., 2005). Different seizure patterns then lead to different parameter paths. The course of each individual seizure is thereby interpreted as a specific path in parameter space.

For focal-onset seizures, the local onset, spreading and (self-)termination of the seizure calls for an additional description of the spatial features. Imaging of the spatio-temporal evolution of epileptic activity in an acute model of focal-onset seizure shows rhythmic activity to result from complex spatio-temporal patterns that show behavior reminiscent of complex wave propagation in excitable media (Viventi et al., 2011). The mammalian neocortex has been experimentally shown to possess some properties of an excitable medium (Ferezou et al., 2007), and the question of generation of spatio-temporal epileptic rhythm can therefore be addressed in that context. Excitable media are a class of models that support a large number of spatio-temporal patterns. A suggestion from nonlinear dynamics is thus to investigate coupled neural mass models as an approximation of an excitable medium.

One study considered spatially coupled compartments in the vicinity of abnormal (epileptic) spiking and investigated its response to local stimulation (Goodfellow et al., 2012a). If spatial heterogeneity was included (modeled by compartments with abnormally decreased inhibition), the model responded to single pulse stimuli within or near the heterogeneous region with abnormal rhythmic transients. Indeed, abnormal rhythmic responses to single pulse stimuli are observed in human patients during pre-surgical monitoring (Valentin et al., 2005).

The evidence that focal-onset seizures might either originate from a single small localized region (Rosenow and Lüders, 2001) or from distributed abnormal networks (Spencer, 2002) led to the proposal that epileptic rhythms could be self-organised transient phenomena in an excitable medium with differentially distributed tissue heterogeneity (Goodfellow et al., 2012b).

## Summary

**Figure 3:**
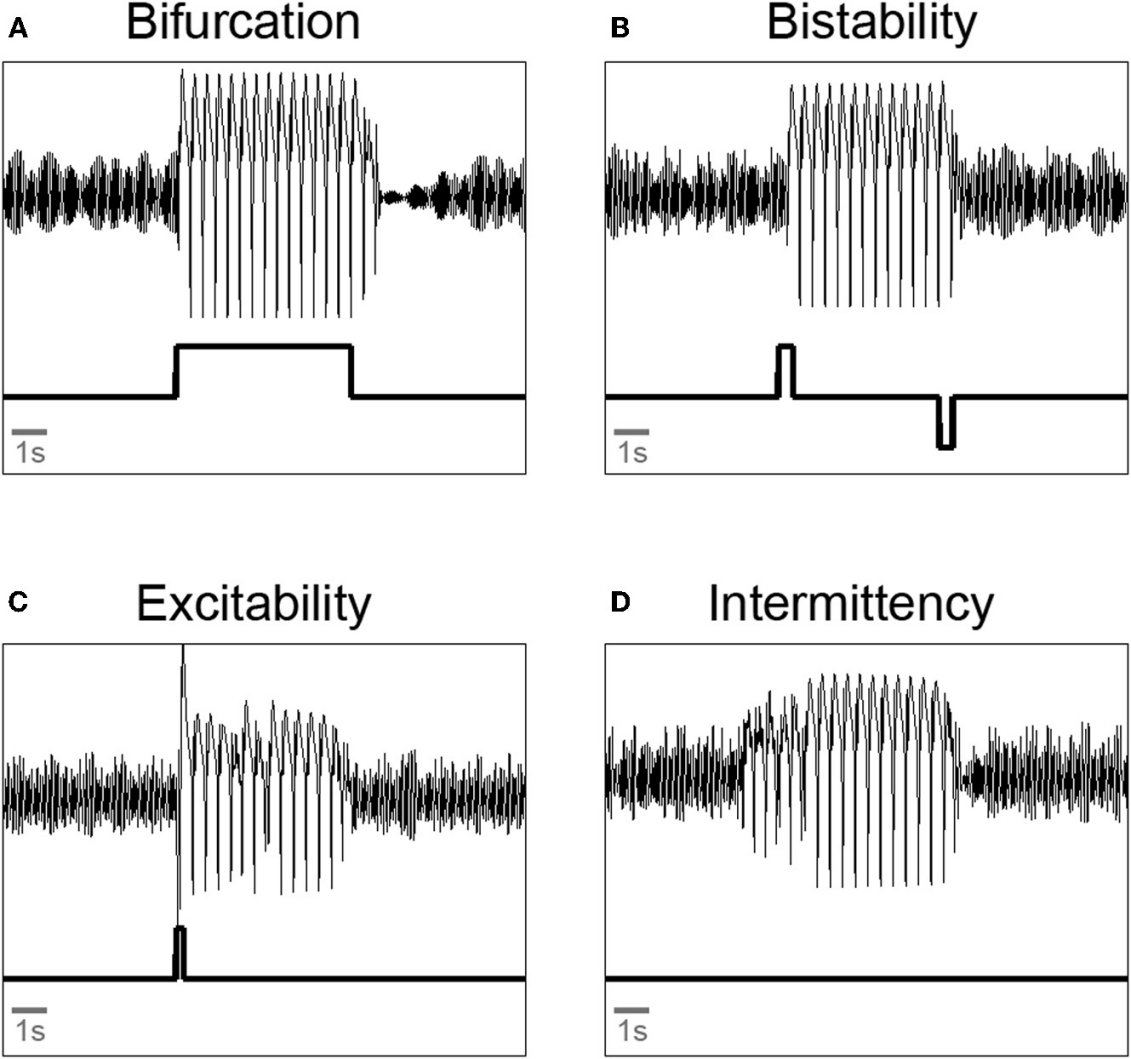
**Illustration of qualitatively different transitions from background oscillations to pathological spike-wave and back again in a neural mass model. (A)** Bifurcation: a parameter is changed such that it crosses a bifurcation point. **(B)** Bistability: two pulse perturbation are applied to start and terminate a seizure. **(C)** Excitability: a single pulse perturbation is applied to induce a seizure. **(D)** Intermittency: parameter setting allows spontaneous transitions into and out of the seizure rhythms. All simulations done with a three compartment version of the extended Jansen-Rit model (Goodfellow et al., 2011). Upper trace: model output. Lower trace: parameter protocol.

Thus, the modeling work suggests novel dynamical mechanisms on the macroscopic scale resulting from known mechanisms of interaction, in this case in the human neocortex. Figure 3 summarizes the findings with exemplary simulations. Epileptic seizure rhythms can be induced from background activity by (a) a change of a parameter to a pathological value (i.e., crossing a bifurcation point) for the length of the seizure (Figure 3A); (b) one perturbation to induce the seizure rhythm and another perturbation to terminate it in the case of a bistability of background and seizure rhythm (Figure 3B); (c) a single perturbation in the case of a spatio-temporal excitable system (Figure 3C); and (d) no perturbation at all in the case of spatio-temporal intermittency (Figure 3D). The latter two cases have only been observed in spatially extended neural mass models. The parameter changes corresponding to cases (a), (b) and (c) could be due to random fluctuations of parameters on a slow (a) or fast (b and c) time scale, respectively. In case (d) seizure generation is spontaneous.

For the future we expect that neural population models can be adjusted to include spatial heterogeneities as determined by imaging data from individual patients, e.g., from fMRI (Bojak et al., 2011). Additionally, the impact of network topology is incompletely understood. However, recently structural connectivities derived from diffusion tensor imaging of normal subjects and epileptic patients are becoming available and can be incorporated in modeling studies (Taylor et al., submitted). The spatio-temporal output can then be fitted to EEG data on the clinically relevant scales and, if optimized for performance, could in principle run in real time with continuous input of patient EEG to detect specific abnormalities, see e.g., (Baier et al., 2000) and (Chernihovskyi et al., 2005). Furthermore, *in silico* experiments allow the design of perturbation protocols to suppress seizure activity using e.g., electrical stimulation from implanted electrodes (Kalitzin et al., 2010).

To conclude, spatio-temporal neural mass models are a missing link between experimental neurophysiological findings and clinical manifestations of epilepsy. They offer a better mechanistic interpretation of the multiple factors influencing the complex pathophenotypes across the epilepsies.

### Conflict of interest statement

The authors declare that the research was conducted in the absence of any commercial or financial relationships that could be construed as a potential conflict of interest.
